# The susceptibility of shi drum juveniles to betanodavirus increases with rearing densities in a process mediated by neuroactive ligand–receptor interaction

**DOI:** 10.3389/fimmu.2024.1304603

**Published:** 2024-06-12

**Authors:** José María García-Beltrán, Carolina Johnstone, Marta Arizcun, Alberto Cuesta, Montse Pérez, Elena Chaves-Pozo

**Affiliations:** ^1^ Physiology and Welfare of Marine Species Group (PHYSIS), Centro Oceanográfico de Murcia, Instituto Español de Oceanografía (COMU-IEO), Consejo Superior de Investigaciones Científicas (CSIC), Murcia, Spain; ^2^ Immunobiology for Aquaculture Group, Department of Cell Biology and Histology, Faculty of Biology, University of Murcia, Murcia, Spain; ^3^ Physiology and Welfare of Marine Species Group (PHYSIS), Centro Oceanográfico de Málaga, Instituto Español de Oceanografía (COMA-IEO), Consejo Superior de Investigaciones Científicas (CSIC), Málaga, Spain; ^4^ Centro Oceanográfico de Vigo, Instituto Español de Oceanografía (COV-IEO), Consejo Superior de Investigaciones Científicas (CSIC), Vigo, Spain

**Keywords:** *Umbrina cirrosa*, nodavirus, welfare, immune response, stocking densities, pathogenesis, immune-neuroendocrine interactions

## Abstract

Nervous necrosis virus (NNV) is one of the greatest threats to Mediterranean aquaculture, infecting more than 170 fish species and causing mortalities up to 100% in larvae and juveniles of susceptible species. Intensive aquaculture implies stressed conditions that affect the welfare of fish and their ability to fight against infections. In fact, a higher susceptibility to NNV has been related to poor welfare conditions. In order to analyze the physiological link between stressed conditions and increased susceptibility to NNV, as well as its possible role in the pathogenesis of this disease, we reared shi drum (*Umbrina cirrosa*) juveniles (30.7 ± 3.10 g body weight), which are expected to be asymptomatic upon NNV infection, at three stocking densities (2, 15, and 30 kg/m^3^) for 27 days and subsequently challenged them with NNV. We firstly characterized the stressed conditions of the specimens before and after infection and recorded the mortalities, demonstrating that stressed specimens reared at 30 kg/m^3^ suffered mortalities. However, the viral loads in different tissues were similar in all experimental groups, allowing horizontal and vertical transmission of the virus from asymptomatic specimens. All of these data suggest that shi drum tolerates wide ranges of culture densities, although high densities might be a setback for controlling NNV outbreaks in this species. In an attempt to understand the molecular pathways orchestrating this susceptibility change in stressed conditions, we performed a transcriptomic analysis of four tissues under mock- and NNV-infected conditions. In addition to the modification of the exceptive pathways such as cell adhesion, leukocyte migration, cytokine interaction, cell proliferation and survival, and autophagy, we also observed a heavy alteration of the neuroactive ligand–receptor pathway in three of the four tissues analyzed. Our data also point to some of the receptors of this pathway as potential candidates for future pharmacological treatment to avoid the exacerbated immune response that could trigger fish mortalities upon NNV infection.

## 1 Introduction

Aquaculture is one of the world’s fastest growing food sectors and the main fish supplier to the world’s population (1). For this reason, the diversification of aquaculture species with economic potential favors the sustainability of aquaculture and is becoming increasingly important in the development of this production sector (2, 3). Among the new fish species, shi drum (*Umbrina cirrosa*) is an ideal candidate with high economic and nutritional value, particularly in the Mediterranean area (4, 5). Thus, shi drum presents high growth rate, elevated market value, good meat quality, and great adaptability to culture conditions (4–6), being a promising candidate for Mediterranean aquaculture diversification. However, and with regard to the FAO annual reports, shi drum was cultivated in low quantities in Italy in 2013 (7) and, according to some publications, in Greece in the 1990s (8). The fact that the industrial culture of shi drum has not been consolidated yet could be due to some gaps on the knowledge about its biology, to technical problems, or to inadequate market promotion policies that dissuade enterprises to produce this species, among others (3). Although several aspects of shi drum biology have been studied since the 1990s, such as its reproduction, digestive system development, dietary requirements, and growth performance (9), its immunity or stress has only recently started to be assessed (9–11).

Stocking density is one of the main factors that lead to chronic stress responses in fish (12). In this sense, high stocking densities typically have adverse effects on the growth, health, and general welfare of fish (13, 14), while low stocking densities could reduce feed competition, resulting in a decrease in feed intake and growth (14). In aquaculture facilities, chronic stress could be identified through the study of operational welfare indicators (OWIs), which are parameters specific to each species and farming system, related to health from the functional approach to wellbeing, and include both observational measures and water quality and biological parameters (15). The identification of chronic stress is mandatory as it can cause the development of diseases (16). In particular, stress can trigger infectious diseases (17). At present, the infectious diseases with the highest threat to aquaculture are those caused by viruses (17). Among them, the nervous necrosis virus (NNV; family Nodaviridae, genus *Betanodavirus*) is one of the most important marine fish viruses, causing the viral encephalopathy and retinopathy (VER) disease (18, 19). NNV is a non-enveloped virus with a spherical shape and icosahedral symmetry and a genome composed of two single-stranded, positive-sense RNA molecules (20) showing horizontal and vertical transmission (18, 19, 21). Betanodaviruses are classified into four genotypes: RGNNV (red-spotted grouper nervous necrosis virus), SJNNV (striped jack nervous necrosis virus), BFNNV (barfin flounder nervous necrosis virus), and TPNNV (tiger puffer nervous necrosis virus) (21). They are capable of infecting more than 170 marine species, including some of great commercial value (21). Shi drum is susceptible to NNV as severe natural outbreaks of RGNNV have been reported in the Mediterranean Sea in both wild and farmed specimens (5, 22–24). To our knowledge, only the viral isolate It/24/Sdr, which corresponds to the RGNNV genotype, was obtained from a wild shi drum specimen captured in Italian marine areas in 1995 (25). In the Adriatic Sea, however, wild shi drum specimens have been included in a multiple species test, but none of them resulted positive, although other fish species did (26). The rest of the reports that detected NNV in shi drum analyzed farmed specimens (5, 8, 27).

Under experimental conditions, shi drum juveniles of 10 g body weight (bw) were found susceptible to all NNV genotypes (RGNNV, SJNNV, BFNNV, and TPNNV), although the signs of the disease and lesions varied depending on the genotype (9). In general, VER disease is characterized by neurological abnormalities (e.g., erratic swimming and spiral movements with the belly up) and a distinct vacuolization of the nerve tissue (brain and retina) (19, 28). VER disease has been detected in larval and early juvenile stages in most of the species (29), although chronic subclinical infections occurred directly proportionally to the age and weight of the fish (18, 19, 21, 30). In addition to host age or weight, other factors related to stressed conditions, such as suboptimal feed, water quality, or crowding, have been reported to influence NNV infection, leading to mortalities in the case of subclinical infection or increasing mortalities in acute infections (30).

The integration of all the physiological functions upon infection is orchestrated by the immune–neuroendocrine system, which determines either the survival of the specimen or the appearance of the disease, as well as the subsequent mortality in different host and external conditions (31). However, in fish, this system is not completely understood in part due to its great complexity, amplified by genome duplication events occurring during fish evolution, but also due to epigenomic reprogramming that has been linked to environmental clues in marine fish (32). In order to design efficient tools to combat NNV infection, it is important to understand the epidemiology and the pathogenic mechanism of the virus and its association with host physiology and welfare. The stress status in fish can be evaluated through hormones (cortisol), secondary metabolites (glucose and lactate), and the imbalance in the physiological responses such as growth performance, key humoral innate immune activities, or the antioxidant system (33) in different tissues such as the serum, skin mucus, brain, head kidney, spleen, or liver (34, 35). During stress conditions, there is an imbalance in the production of reactive oxygen species (ROS) and its elimination by the antioxidant system, resulting in high concentrations of ROS that can lead to oxidative stress and make fish susceptible to physiological and metabolic diseases (36). Taking all of these into account, the aim of the study was to assess the physiological status of fish reared at three different densities and the association of crowding and stress with susceptibility to NNV. The stress and the welfare status of the fish were characterized using well-known stress parameters and innate immune responses in the serum and skin mucus. The skin mucus is considered a noninvasive sampling tissue and is continuously produced, with functions including the prevention of the adherence of pathogens to underlying tissues and the provision of a medium in which antimicrobial mechanisms can act (37). In addition, the levels of cortisol in water were included in order to evaluate this new noninvasive stress indicator. Upon infection, the stress indicators were analyzed, as well as the mortality rates and the persistence of the virus in different tissues of surviving fish. In the acute viral infection phase, differential gene expression was assessed using a transcriptomic approach in four tissues: the viral target tissue, the brain (and also the first step in most endocrine regulatory axes) (38), and three immune-related tissues—the spleen, the head kidney, and the liver—to explore the immune–neuroendocrine interactions that might link stress with NNV susceptibility in order to identify potential pharmacological targets for further treatment development.

## 2 Materials and methods

### 2.1 Animals

Healthy juveniles of shi drum (*U. cirrosa*) (30.7 ± 3.10 g bw) obtained from natural spawns of culture broodstock (39) were bred at the facilities of the Centro Oceanográfico de Murcia, Instituto Español de Oceanografía (COMU-IEO), CSIC, as described elsewhere (4). Fish were reared with an open-flow natural seawater system (38‰ salinity), suitable aeration to maintain dissolved oxygen above 6.5 mg/L, filtration systems, natural temperature (21.47 ± 1.09°C) and photoperiod, and a culture density of 9 kg/m^3^. Ammonia (<0.1 mg/L), nitrite (<0.2 mg/L), and nitrate (<50 mg/L) were determined once weekly.

Handling of the specimens was always performed under the Guidelines of the European Union Council (2010/63/UE) and the Bioethical Committees of the IEO (REGA code ES300261040017) and with approval of the Ministry of Water, Agriculture and Environment of the Autonomous Community Region of Murcia (permit no. A13211203).

### 2.2 Experimental design and sample collection

Based on the aim of this study, fish reared at 9 kg/m^3^ were randomly assigned and divided into nine tanks of 0.25 m^3^ capacity with an initial density of 2 kg/m^3^ (*n* = 15 fish/tank), 15 kg/m^3^(*n* = 118 fish/tank), or 30 kg/m^3^ (*n* = 235 fish/tank) at the beginning of the experiment and a final density of 1 kg/m^3^ (*n* = 6 fish/tank), 17 kg/m^3^ (*n* = 109 fish/tank), or 35 kg/m^3^ (*n* = 226 fish/tank) at the end of the experiment. Each condition was performed in triplicate tanks. The fish were fed *ad libitum* with a commercial pellet diet (Skretting, Stavanger, Norway), with a maximum intake of 2.8% of their biomass for 27 days. After 7, 21, and 27 days from the start of the experiment, three fish from each tank (*n* = 9 fish/experimental group) were sampled. Before starting the collection of specimens, water samples from each tank (100mL/tank) were obtained at each sampling time and stored at −80°C until use. Thereafter, fish (*n* = 3 fish/tank) were captured and placed in an anesthesia bath with 40 µL/L of clove oil. Once the fish reached the loss of reflex reactivity stage of anesthesia, skin mucus samples were collected using a method previously described (40). The skin mucus was vigorously shaken and centrifuged (400 × *g*, 10 min at 4°C). The supernatant was then collected and stored at −80°C until use. After measuring the bw and length of each fish, they were completely bled. The blood samples were left to clot at 4°C for 4 h. Subsequently, the serum was obtained after centrifugation (10,000 × *g*, 10 min at 4°C) and stored at −80°C until use.

On day 27, 10 fish from each tank (*n* = 30 fish/experimental group) were relocated in the infection facilities in 200-L tanks in a close recirculated seawater flux (38‰ salinity), with a 12-h light/12-h dark photoperiod and 27 ± 1°C controlled temperature for NNV challenge. The animals were fed *ad libitum* daily with a commercial pellet diet (Skretting). A control group was established with fish of the 30-kg/m^3^ group, which were mock-infected. Infected fish (*n* = 6/experimental group) were sampled 4 and 18 days post-infection (dpi). The specimens were anesthetized as described previously. Samples of the eye, brain, liver, spleen, and head kidney were collected at 4 dpi and those of the brain and gonad at 18 dpi. The tissue samples were immersed on a DNA/RNA shield (Zymo Research, Irvine, CA, USA) and stored at −80°C until further processing for gene expression analysis.

### 2.3 Nodavirus infection

NNV (genotype RGNNV, strain It/411/96) was propagated in the E-11 cell line [17]. NNV stocks were titrated and the viral dilution infecting 50% of the cell cultures (TCID_50_) calculated (41). For NNV infection, fish from all experimental groups were intramuscularly injected with 100 μL of the viral suspension (5.6 × 10^6^ TCID_50_/mL) or with phosphate-buffered saline (PBS) in the case of the mock-infected group (control). The mortality and clinical signs of infection were recorded daily and the percentage of survival determined. Surviving fish were considered as those fish without any signs of disease during the infection trial or those able to overcome signs of disease within 3 days. In contrast, susceptible fish were those that died during the trial or showed signs of disease for three consecutive days, which were then humanely euthanized using baths containing 40 μL/L of clove oil according to the guidelines on the care and use of fish (42).

### 2.4 Growth performance

Growth was monitored by obtaining the specific growth rate (SGR), which was calculated as: [(ln final weight − ln initial weight)/number days] × 100. Moreover, the condition factor (CF) following Fulton’s *K*-index was calculated as: (bw − lenght^3^) × 100.

### 2.5 Plasmatic and skin mucus analysis

The total protein concentration in skin mucus samples was determined using the dye binding method of Bradford (43).

Peroxidase activity was measured as the ability of the samples to oxidate the substrate 3,3′,5,5′-tetramethylbenzidine hydrochloride (TMB; Sigma, St. Louis, MO, USA) in the presence of hydrogen peroxide according to a protocol previously described (44) using 5 µL of serum or 10 µL of skin mucus. Wells with buffer but not samples were used as blanks. One unit was defined as the amount of activity producing an absorbance change of 1, and the activity was expressed as units per milliliter of serum or units per microgram protein of skin mucus samples.

Protease activity was determined as the percentage of hydrolysis of azocasein (Sigma) using a modified protocol previously described (45) and 10 µL of serum or 30 µL of skin mucus. Proteinase K (2 mg/mL; AppliChem, Darmstadt, Germany) or PBS instead of samples was used as a positive control (100% of activity) or a negative control (0% of activity), respectively. The percentage of protease activity for each sample was calculated as the percentage of activity of the positive control. The results were expressed as percent of activity.

The antiprotease activity in 10 μL of serum or 30 µL of skin mucus was determined as the ability of the samples to inhibit the activity of proteinase K using a modified protocol previously described (46). The blank was prepared by replacing the samples and proteinase K (2 mg/mL) for PBS (no protease activity), while PBS instead of samples was used as the negative control (100% of activity). The percentage of inhibition of proteinase K activity for each sample was calculated as: [100 − (% of protease activity)]. The results were expressed as percent of activity.

Lysozyme activity in 100 µL of serum (diluted 1:2) or 100 µL of skin mucus samples was measured using a modified turbidimetric method previously described (47) and was based on the lysis of 0.3 mg/mL of freeze-dried *Micrococcus lysodeikticus* (Sigma). Changes in the absorbance at 450 nm were measured immediately every 30 s for 30 min at 25°C in a plate reader (MultiskanGo; Thermo Fisher Scientific, Waltham, MA, USA). One unit of lysozyme activity was defined as a reduction in the absorbance of 0.001/min. The lysozyme units were obtained from a standard curve ranging from 20 to 0 µg/mL made with hen egg white lysozyme (HEWL; Sigma). The results were expressed as international units per milliliter of serum or international units per milligram protein of skin mucus samples.

The bactericidal activity of 10 µL of serum or 20 µL of skin mucus samples was determined by evaluating the bacterial growth curves of *Vibrio harveyi* (strain Lg 16/100) using a method previously described (48). The samples replacing bacteria by culture medium were used as negative controls (0% growth and 0% bactericidal activity), while the samples replacing the serum or skin mucus by culture medium were used as positive controls (100% growth or 0% antibacterial activity). Bactericidal activity was calculated as: [100 − (% of bacterial growth)]. The results were corrected using the absorbance measured in each sample at the initial time point and were expressed as percent of activity.

The glucose levels in 4 µL of skin mucus samples were determined using the Glucose-HK enzymatic kit (Spinreact, Girona, Spain) following the manufacturer’s instructions. The positive control was established using 4 µL of glucose standard (100 mg/dL) instead of samples, while wells containing only buffer were used as blanks. The results were expressed as milligrams per deciliter.

The lactate levels in 4 µL of skin mucus samples were determined using the Lactate LO-POD enzymatic kit (Spinreact) following the manufacturer’s instructions. Positive controls were prepared using 4 µL of lactate standard (10 mg/dL) instead of samples, while wells containing only buffer were used as blanks. The results were expressed as milligrams per deciliter.

The cortisol from the water samples was extracted using a modified protocol previously described (49). Briefly, frozen water samples were thawed and filtered (F1091–130F). The samples were run through extraction cartridge C18 (SEP-PAK; Waters, Milford, MA, USA) following the manufacturer’s instructions. Steroids were eluted with 5 mL of 100% methanol. Methanol was evaporated by incubation at 35°C and the steroids reconstituted in 1 mL reaction buffer (RB) [0.1 M phosphate–potassium buffer (PPB), 0.1% bovine serum albumin (BSA), 1 mM ethylenediaminetetraaceticacid (EDTA), 0.4 M NaCl, and 1.5 mM sodium azide (NaN_3_), pH 7.4].

The cortisol levels in 5 µL of serum and 20 µL of skin mucus samples or extracted water samples were determined using the cortisol competitive human ELISA kit (Invitrogen, Carlsbad, CA, USA) following the manufacturer’s instructions. A cortisol standard curve from 3,200 to 50 pg/mL serial dilutions was established, and the samples and standards were analyzed in duplicate. Wells with assay buffer instead of sample and without the cortisol antibody were used as blanks. The results were expressed as nanograms per milliliter.

The total antioxidant activity of the skin mucus samples was analyzed using the 2,2′-azino-bis-3-(ethylbenzothiazoline-6-sulfonic acid) (ABTS) method previously described (50), which was based on the ability of the antioxidants in the sample to reduce the radical cation of ABTS, as determined by the decoloration of ABTS+, and by measuring the quenching of the absorbance at 730 nm. Wells containing only PBS were used as blanks. This activity was calculated by comparing the values of the sample with a standard curve of ascorbic acid and was expressed as ascorbic acid equivalents (in millimoles) per milligram protein.

### 2.6 Viral gene expression in NNV-infected shi drum tissues

Total RNA was isolated from fragments of frozen tissues (the brain from 4 and 18 dpi fish; the eye, liver, spleen, and head kidney from 4 dpi fish; and the gonad from 18 dpi fish) preserved in DNA/RNA Shield™ (Zymo Research) using the Quick-RNA™ MiniPrepPlus Kit (Zymo Research) following the manufacturer’s instructions. The SensiFast™ cDNA synthesis kit (Bioline Meridian Life Science, Memphis, TN, USA) was used to synthesize cDNA by mRNA reverse transcription according to the manufacturer’s instructions. Subsequent real-time PCR was performed with the AriaMx real-time PCR system (Agilent, Santa Clara, CA, USA) using the PowerUp™ SYBR™ Green Master Mix (Applied Biosystems, Foster City, CA, USA) and viral NNV coat protein (CP) specific primers (**Supplementary Table S1**). The reaction mixtures were incubated for 10 min at 95°C, followed by 40 cycles of 15 s at 95°C, 1 min at 60°C, and a final 15 s at 95°C, 1 min at 60°C, and 15 s at 95°C. For each mRNA, gene expression was corrected by the expression of the *beta-actin* (*actb*) gene in each sample and was expressed as 2^−Δ^ *^C^* ^t^, where Δ*C*_t_ was determined by subtracting the *C*_t_ value of *actb* from that of the target (51).

### 2.7 RNA isolation, library construction, and sequencing

We next analyzed the transcriptome of the viral target tissue (brain) and the immune-related tissues (spleen, head kidney, and liver) of the mock- and NNV-infected fish groups, in which higher mortalities were recorded (reared at 30 kg/m^3^ prior to infection). Total RNA was extracted from each sample using the Trizol reagent (Invitrogen) according to the manufacturer’s specifications. The preparation of RNA libraries and transcriptome sequencing were conducted by Novogene Co., Ltd. (Cambridge, UK). mRNA was purified from total RNA using poly-T oligo-attached magnetic beads. After fragmentation, the first-strand DNA was synthesized using random hexamer primers, followed by the second-strand cDNA synthesis. Libraries were ready after end repair, A-tailing, adapter ligation, size selection, amplification, and purification. Thereafter, the libraries were checked with Qubit and real-time PCR for quantification and bioanalyzer to determine the size distribution. Quantified libraries were sequenced on the Illumina platform (Instrument HWI-ST1276). The original raw data from the Illumina platform were transformed to sequenced reads through base calling and were recorded in a FASTQ file (52). In this case, in the absence of a reference genome, clean reads were assembled using Trinity (53). Subsequently, the CORSET software (54) was used to remove the redundancies from the Trinity results. Finally, BUSCO was used to attempt to provide a quantitative assessment of completeness in terms of the expected gene content of the transcriptome.

### 2.8 Bioinformatics analysis of transcriptome data

#### 2.8.1 Gene functional annotation

In order to obtain a comprehensive gene functional annotation, seven databases were used: NCBI non-redundant protein sequences (Nr), NCBI nucleotide sequences (Nt), Protein family (Pfam), Cluster of Orthologous Groups of Protein/Eukaryotic Orthologous Groups (KOG/COG), Swiss-Prot, Kyoto Encyclopedia of Genes and Genomes (KEGG), and Gene Ontology (GO). CDS prediction was performed in two steps. Firstly, BLAST was used to align the unigene sequences to NR and Swiss-Prot; if matched, the CDS was translated into peptide sequences. If there was no match, TransDecoder was used to predict the coding regions and to determine the sequence direction. The AnimalTFDB database was used to perform the transcription factor analysis.

#### 2.8.2 Gene expression analysis

As the reference transcriptome, the *de novo* shi drum filtered by Corset was used. RSEM (55) was used to quantify the expression levels. The read count for each sample was converted into FPKM (fragments per kilobase of transcript per million mapped reads) values. To compare the gene expression levels under different conditions, the FPKM distribution diagram was used. To reveal differences in the gene expression between samples and the repeatability of the experiments (by comparison of replicates), the square of Pearson’s correlation coefficient was calculated.

#### 2.8.3 Differential expression analysis

Read counts from the gene expression level analysis were normalized with DESeq2 (56). A negative binomial distribution was used as a model for the estimation of *p*-values; for calculation of the false discovery rate (FDR), the Benjamini–Hochberg (BH) method was applied [log2(FoldChange), *p*_adj_ < 0.05]. Cluster analysis was performed to discover genes with similar expression patterns under various experimental conditions.

#### 2.8.4 GO enrichment analysis

In order to determine which biological functions or pathways are significantly associated with differentially expressed genes (DEGs), GO enrichment analysis was performed with GOseq and topGO ACG plotting was done with topGO. KEGG annotates a gene to the pathway level and was applied using KOBAS, while protein–protein interaction analysis was performed with the NCBI BLAST 29.0 software.

### 2.9 Quantitative real-time PCR validation for mRNA expression

The sequences of four potential reference genes were retrieved from transcriptome data and were used to design specific primers using the NCBI Primer-BLAST software (**Supplementary Table S1**). Real-time PCR reactions were run as described above. To confirm the specificity of each primer pair, melting curve analysis of the amplified products was performed. Negative controls with no template were always included in all the reactions.

### 2.10 Statistical analyses

The results from the growth performance rates and plasmatic and skin mucus analysis, as well as the gene expression, were expressed as the mean ± SEM. Normality of the variables was confirmed with the Shapiro–Wilk test, while homogeneity of variance was assessed using the Levene test. Two-way ANOVA was performed to determine differences between rearing densities and time point. Upon infection, differences between the mock-infected and infected specimens were analyzed according to the Student’s *t*-test. Survival was determined by the Kaplan–Meier method, and statistical differences were studied using a log-rank (Mantel–Cox) test. The significance level was 95% in all cases (*p* ≤ 0.05). All data were analyzed using SPSS for Windows ® (version 15.0; SPSS Inc., Chicago, IL, USA). Some of the figures were drawn using the freely available SRplot web server (https://www.bioinformatics.com.cn).

## 3 Results

### 3.1 Increasing the rearing density altered the shi drum stress parameters while scarcely affecting growth performance

**Figure 1:**
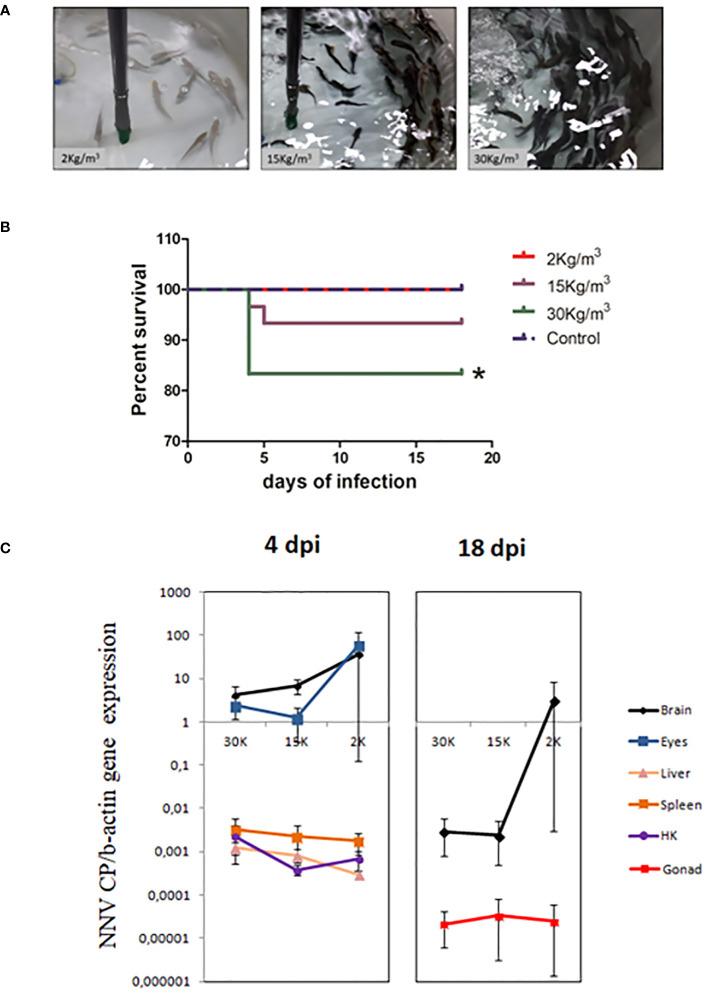
Juvenile shi drum specimens became susceptible to nervous necrosis virus (NNV) under stressed conditions. **(A)** Photographs showing the skin color of shi drum juveniles reared at 2, 15, or 30 kg/m^3^ density. **(B)** Survival percentage of shi drum juveniles reared at 2, 15, or 30 kg/m^3^ density for 27 days and then infected with 5.6 × 10^5^ TCID_50_/mL of NNV. **(C)** Relative expression of the viral capsid protein gene in several tissues of shi drum juveniles reared at different densities for 27 days and then infected with NNV. *dpi*, days post-infection. Asterisks denote statistically significant differences between control and infected groups accordingly with a Log-ranked (Mantel-Cox) test.

Scarce differences were observed in growth performance when fish reared at different densities were compared at the same time point. After 27 days of culture, the fish farmed at 2 kg/m^3^ showed higher SGR than those farmed at 15 kg/m^3^ (**Table 1**). The opposite results were observed for the stress parameters (**Table 2**). Thus, the serum cortisol levels were higher at all time points in fish reared at 15 and 30 kg/m^3^ than in those reared at 2 kg/m^3^. The same pattern was observed in the skin mucus cortisol levels on days 7 and 27 and in skin mucus glucose on day 27 (**Table 2**). Interestingly, when fish reared at the same density were compared through time, statistically significant differences were observed in the glucose levels of skin mucus from 21 days onwards at all rearing densities. Moreover, increases in the ABTS activity through time were observed at the rearing densities of 15 and 2 kg/m^3^ (**Table 2**). The skin color of fish reared at 15 and 30 kg/m^3^ was dark at the end of the experiment (**Figure 1A**). Interestingly, the bactericidal and lysozyme activities and the cortisol levels from the serum and skin mucus were positively correlated, as well as glucose and cortisol or glucose and lactate from the skin mucus. In contrast, a negative correlation was observed between the skin mucus glucose and the ABTS levels (**Table 3**).

**Table 1:** Growth performance data.

	7 days			21 days			27 days		
	2 kg/m^3^	15 kg/m^3^	30 kg/m^3^	2 kg/m^3^	15 kg/m^3^	30 kg/m^3^	2 kg/m^3^	15 kg/m^3^	30 kg/m^3^
SGR (%)	0.94 ± 1.04	−0.65 ± 1.08	1.34 ± 1.29	1.32 ± 0.35	0.28 ± 0.27	0.28 ± 0.45	1.19 ± 0.24a	0.46 ± 0.16b	0.68 ± 0.26ab
CF (%)	1.24 ± 0.02	1.30 ± 0.03	1.33 ± 0.05	1.20 ± 0.01	1.30 ± 0.04	1.29 ± 0.03	1.20 ± 0.03	1.32 ± 0.03	1.34 ± 0.04

Shown are the specific growth rate (SGR) and condition factor (CF) of shi drum specimens reared at 2, 15, and 30 kg/m^3^ for 7, 21, and 27 days. Lowercase letters denote statistically significant differences between different rearing densities at the same time point (p ≤ 0.05).

**Table 2:** Data of the serum and skin mucus stress parameters of shi drum specimens reared at 2, 15, and 30 kg/m^3^ for 7, 21, and 27 days.

	7 days			21 days			27 days		
	2 kg/m^3^	15 kg/m^3^	30 kg/m^3^	2 kg/m^3^	15 kg/m^3^	30 kg/m^3^	2 kg/m^3^	15 kg/m^3^	30 kg/m^3^
Water cortisol	0.49 ± 0.10	0.55 ± 0.02	0.90 ± 0.55	0.70 ± 0.19	0.72 ± 0.17	0.51 ± 0.14			
Serum cortisol	6.06 ± 3.03a	20.08 ± 7.78b	26.00 ± 15.68b	2.50 ± 1.00a	9.44 ± 2.55b	28.81 ± 14.47b	3.36 ± 0.83a	8.64 ± 2.24b	13.06 ± 7.81b
Skin mucus cortisol	1.31 ± 0.39a	3.08 ± 0.58b	3.51 ± 0.96b	2.01 ± 0.32	1.33 ± 0.50*	2.88 ± 0.89	0.65 ± 0.06a	1.59 ± 0.40b	2.13 ± 0.79b
Skin mucus glucose	12.42 ± 1.46	12.46 ± 1.46	12.62 ± 1.27	7.08 ± 0.53*	6.38 ± 0.44*	6.91 ± 0.94*	5.47 ± 0.66a*	9.00 ± 1.00b*	8.97 ± 1.25b*
Skin mucus lactate	1.02 ± 0.21	1.24 ± 0.18	1.39 ± 0.20	0.77 ± 0.15	0.90 ± 0.23	1.09 ± 0.22	0.68 ± 0.28	0.87 ± 0.18	1.35 ± 0.26
Skin mucus ABTS	3.63 ± 0.23	2.64 ± 0.14	3.32 ± 0.48	4.57 ± 0.82	8.59 ± 2.02*	4.94 ± 1.13	6.44 ± 0.89*	5.11 ± 0.56	4.24 ± 0.37

Lowercase letters denote statistically significant differences between different rearing densities at the same time point. Asterisk denotes statistically significant differences between time points at the same rearing condition (p ≤ 0.05).

**Table 3:** Correlation observed in the serum and skin mucus immune and stress parameters.

	Serum lysozyme	Serum bactericidal	Serum cortisol	Skin mucus peroxidase	Skin mucus lysozyme	Skin mucus bactericidal	Skin mucus cortisol	Skin mucus glucose	Skin mucus lactate	Skin mucus ABTS
Serum lysozyme	1.000	−0.044	−0.138	0.120	**0.830**	−0.111	−0.204	−0.153	−0.369	0.187
		0.789	0.429	0.463	**0.000**	0.515	0.319	0.352	0.021	0.331
Serum bactericidal		1.000	−0.292	−0.100	0.017	**0.484**	−0.277	−0.120	0.054	−0.006
			0.035	0.384	0.909	**0.000**	0.045	0.292	0.639	0.963
Serum cortisol			1.000	0.129	−0.077	−0.311	**0.606**	0.189	0.275	−0.185
				0.351	0.668	0.025	**0.000**	0.171	0.044	0.266
Skin mucus peroxidase				1.000	−0.031	−0.006	−0.049	−0.162	−0.136	**0.692**
					0.834	0.959	0.712	0.137	0.216	**0.000**
Skin mucus lysozyme					1.000	−0.048	−0.114	−0.001	−0.189	−0.030
						0.748	0.488	0.994	0.188	0.865
Skin mucus bactericidal						1.000	−0.218	−0.133	0.019	0.221
							0.101	0.233	0.868	0.098
Skin mucus cortisol							1.000	**0.467**	0.356	−0.257
								**0.000**	0.005	0.088
Skin mucus glucose								1.000	**0.564**	−**0.507**
									**0.000**	**0.000**
Skin mucus lactate									1.000	−0.406
										0.001
Skin mucus ABTS										1.000

The number located in the first row of each analyzed activity corresponds to Pearson's correlation coefficient and the number locate in the second row to significant differences (p-value). Values in bold are the parameters that showed correlation. The parameters that did not show any significant correlation were not included.

ABTS, 2,2′-azino-bis-3-(ethylbenzothiazoline-6-sulfonic acid).

### 3.2 Antiprotease and lysozyme activities showed differences at different rearing densities and through time

No differences were observed in the serum or skin mucus peroxidase, protease, and bactericidal activities, nor in the serum lysozyme activity of fish farmed at different densities at any assayed time (**Table 4**).

**Table 4:** Data of the serum and skin mucus innate immune parameters of shi drum specimens reared at 2, 15, and 30 kg/m^3^ for 7, 21, and 27 days.

	7 days			21 days			27 days		
	2 kg/m^3^	15 kg/m^3^	30 kg/m^3^	2 kg/m^3^	15 kg/m^3^	30 kg/m^3^	2 kg/m^3^	15 kg/m^3^	30 kg/m^3^
Serum peroxidase	153.00 ± 16.15	186.31 ± 27.94	152.28 ± 30.26	198.65 ± 40.02	149.18 ± 23.79	189.30 ± 31.79	108.27 ± 20.10	149.70 ± 21.83	107.79 ± 18.40
Skin mucus peroxidase	10.83 ± 0.48	11.46 ± 0.97	13.39 ± 1.40	10.03 ± 0.77	14.84 ± 2.26	13.60 ± 1.72	13.86 ± 1.95	14.89 ± 1.13	13.50 ± 1.15
Serum protease	0.86 ± 0.14	1.18 ± 0.13	0.86 ± 0.21	0.82 ± 0.11	1.15 ± 0.17	0.95 ± 0.19	0.79 ± 0.15	0.38 ± 0.13	0.67 ± 0.10
Skin mucus protease	0.12 ± 0.03	0.39 ± 0.20	0.18 ± 0.05	0.21 ± 0.06	0.12 ± 0.02	0.36 ± 0.24	0.14 ± 0.04	0.54 ± 0.29	0.34 ± 0.16
Serum antiprotease	12.20 ± 0.93	9.97 ± 0.75	11.60 ± 0.80	10.08 ± 0.82	10.70 ± 1.30	8.55 ± 1.01*	11.91 ± 0.82a	8.68 ± 1.07b	9.90 ± 0.52ab
Skin mucus antiprotease	2.07 ± 0.27a	2.98 ± 0.29ab	3.30 ± 0.33b	2.32 ± 0.20	2.20 ± 0.22*	2.38 ± 0.21*	1.85 ± 0.33	1.86 ± 0.22*	1.89 ± 0.31*
Serum lysozyme	1,018.42 ± 48.32	1,210.83 ± 183.28	1,164.10 ± 103.27	968.28 ± 72.19	1,190.50 ± 78.37	1,230.77 ± 82.93	1,001.42 ± 70.92	1,036.09 ± 215.57	974.36 ± 87.00
Skin mucus lysozyme	15.67 ± 6.22	6.54 ± 3.04	27.65 ± 7.73	26.70 ± 7.87a	129.29 ± 37.60b	45.22 ± 8.90ab*	56.81 ± 6.55a*	23.31 ± 5.15b*	21.61 ± 6.64b*
Serum bactericidal	17.38 ± 3.06	16.70 ± 3.74	10.78 ± 3.04	13.87 ± 2.18	12.94 ± 2.37	9.95 ± 2.20	17.35 ± 2.13	11.65 ± 3.73	13.89 ± 2.65
Skin mucus bactericidal	8.74 ± 1.45	10.04 ± 2.55	10.02 ± 1.73	12.29 ± 1.81	14.92 ± 1.80	10.34 ± 2.14	11.56 ± 1.97	10.77 ± 2.73	12.72 ± 3.39

Lowercase letters denote statistically significant differences between different rearing densities at the same time point. Asterisk denotes statistically significant differences between time points at the same rearing condition (p ≤ 0.05).

In the case of antiprotease activity, fish reared at 2 kg/m^3^ for 27 days showed higher serum levels than fish reared at higher densities, which was significantly lower in fish reared at 15 kg/m^3^. In addition, fish reared at 30 kg/m^3^ for 21 days showed lower levels than the same group at 7 and 27 days (**Table 4**). In the skin mucus, the antiprotease activity levels of fish reared at 2 kg/m^3^ for 7 days were lower than those of fish reared at 30 kg/m^3^ for 7 days (**Table 4**). Interestingly, the antiprotease activity in the skin mucus decreased through time in fish reared at 15 and 30 kg/m^3^. With regard to the lysozyme activity in the skin mucus, fish farmed at 2 kg/m^3^ showed lower or higher levels at 21 or 27 days, respectively, than those farmed at higher densities, which was significantly lower in fish reared at 15 kg/m^3^ for 21 days and in fish reared at 15 and 30 kg/m^3^ for 27 days. In fact, differences in this activity through time were observed in fish reared at 30 kg/m^3^ (**Table 4**).

### 3.3 Mortalities from NNV infection only occurred in fish reared at the highest density

After 27 days of rearing at different densities, the fish were infected with NNV to ascertain any association between rearing densities, stress, and mortalities. As darkness of the skin is an external sign of stress and was observed in fish reared at 30 kg/m^3^ (**Figure 1A**), we used fish from this condition as the mock-infected control in order to exclude any unspecific mortalities due to poor welfare of the fish. After NNV challenge (**Figure 1B**), statistically significant mortalities were only recorded in fish reared at 30 kg/m^3^, although some mortalities also occurred in fish reared at 15 kg/m^3^ (*n* = 5 in 30 kg/m^3^ vs. *n* = 2 in 15 kg/m^3^). In contrast, no mortalities occurred at the lowest density (2 kg/m^3^) or in the control group (**Figure 1B**). Interestingly, all mortalities occurred within the first 5 days of infection (**Figure 1B**), and then the infection appeared to be overcome. Analysis of the transcription levels of the viral CP gene (**Figure 1C**) found no statistically significant differences between fish reared at different densities, either at 4 dpi or at 18 dpi, in any of the tissues analyzed (the brain at 4 and 18 dpi; the eyes, liver, spleen, and head kidney at 4 dpi; and the gonad at 18 dpi). When comparing tissues, lower levels were observed in the liver, spleen, head kidney, and gonad, while the brain and eyes showed more than 100- or 10-fold higher levels at 4 or 18 dpi, respectively (**Figure 1C**).

### 3.4 NNV scarcely alters the stress parameters, but increases the bactericidal activity

With regard to the serum and mucus stress parameters after infection (**Table 5**), only the lactate levels of the skin mucus were significantly increased in the 15-kg/m^3^ infected fish compared with the mock-infected fish. No differences were observed in the peroxidase, protease, and lysozyme activities of the serum or skin mucus after NNV infection at any rearing density (**Table 6**). Interestingly, an inversely proportional relationship between the rearing density and skin mucus antiprotease activity was observed in fish infected with NNV, although only the fish reared at 30 kg/m^3^ showed a statistically significant decrease of this activity when compared with the mock-infected fish (**Table 6**). The bactericidal activity showed significant increases upon infection (**Table 6**). Thus, this activity increased in the skin mucus of fish reared at 2 kg/m^3^ and in the serum and skin mucus of fish reared at 30 kg/m^3^ when compared with the mock-infected fish (**Table 6**).

**Table 5:** Data of the serum and skin mucus stress parameters of shi drum specimens reared at 2, 15, and 30 kg/m^3^ for 27 days and after nervous necrosis virus (NNV) infection.

	Mock-infected	NNV-infected		
	30 kg/m^3^	2 kg/m^3^	15Kg/m^3^	30 kg/m^3^
Serum cortisol	4.35 ± 0.66	4.87 ± 0.70	3.84 ± 0.52	3.44 ± 0.86
Skin mucus cortisol	0.62 ± 0.16	1.64 ± 0.50	2.08 ± 0.67	1.28 ± 0.58
Serum glucose	73.65 ± 20.41	61.95 ± 4.41	111.32 ± 9.70	82.49 ± 12.37
Skin mucus glucose	6.67 ± 0.64	5.56 ± 0.39	6.48 ± 0.63	6.11 ± 0.41
Serum lactate	29.04 ± 8.18	33.12 ± 1.93	37.61 ± 1.64	32.17 ± 3.48
Skin mucus lactate	0.55 ± 0.06	0.74 ± 0.12	1.05 ± 0.17*	1.01 ± 0.31
Skin mucus ABTS	1.64 ± 0.19	1.37 ± 0.13	1.82 ± 0.20	1.57 ± 0.16

Asterisk denotes statistically significant differences between mock-infected and infected specimens (p ≤ 0.05).

**Table 6:** Data of the serum and skin mucus immune parameters of shi drum specimens reared at 2, 15, and 30 kg/m^3^ for 27 days and after nervous necrosis virus (NNV) infection.

	Mock-infected	NNV-infected		
	30 kg/m^3^	2 kg/m^3^	15Kg/m^3^	30 kg/m^3^
Serum peroxidase	122.06 ± 11.67	137.75 ± 14.51	133.48 ± 24.71	138.23 ± 25.51
Skin mucus peroxidase	1.52 ± 0.19	1.26 ± 0.20	1.16 ± 0.22	1.22 ± 0.20
Serum protease	0.88 ± 0.14	1.01 ± 0.11	0.95 ± 0.13	0.93 ± 0.29
Skin mucus protease	0.07 ± 0.02	0.11 ± 0.04	0.16 ± 0.10	0.08 ± 0.04
Serum antiprotease	7.89 ± 0.54	6.37 ± 0.86	7.49 ± 2.07	6.43 ± 1.30
Skin mucus antiprotease	3.25 ± 0.5	2.70 ± 0.44	2.51 ± 0.16	1.70 ± 0.31*
Serum lysozyme	6.44 ± 2.02	12.15 ± 2.77	12.73 ± 3.57	8.65 ± 2.00
Skin mucus lysozyme	38.25 ± 11.14	86.47 ± 20.16	113.16 ± 36.13	66.81 ± 14.62
Serum bactericidal	18.19 ± 3.34	25.25 ± 4.19	25.18 ± 5.17	32.13 ± 5.06*
Skin mucus bactericidal	1.88 ± 1.02	7.93 ± 1.40*	6.97 ± 2.29	9.44 ± 3.17*

Asterisk denotes statistically significant differences between mock-infected and infected specimens (p ≤ 0.05).

### 3.5 The neuroactive ligand–receptor pathway is systemically affected by NNV

**Figure 2:**
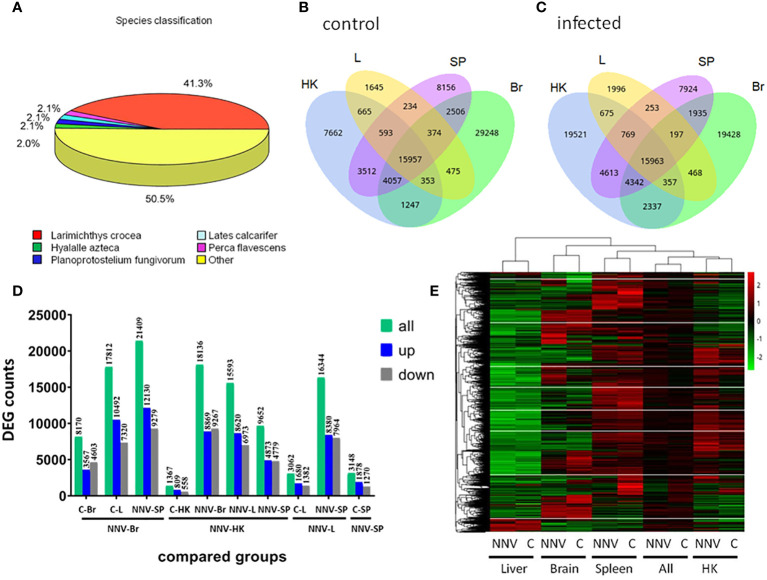
Gene identification and differential expression analysis in mock- and nervous necrosis virus (NNV)-infected head kidney (*HK*), liver (*L*), spleen (*SP*), and brain (*Br*) from shi drum juveniles reared at 30 kg/m^3^ density for 27 days prior to infection. **(A)** Species classification of the unigenes annotated in the *de novo* transcriptome. **(B)** Venn diagram of the expressed genes in HK, L, SP, and Br of mock-infected shi drum juveniles. **(C)**. Venn diagram of the expressed genes in HK, L, SP, and Br of the NNV-infected shi drum juveniles. **(D)** Number of differentially expressed genes (DEGs) in different comparisons performed between mock- and NNV-infected tissues. **(E)** Heatmap showing the cluster analysis of the DEGs in the different tissues analyzed (L, Br, S, and HK) under the mock-infected **(C)** and NNV-infected (*NNV*) conditions. (All) columns represent the expression added in the four tissues analyzed under the mock- and NNV-infected conditions.

The response on day 4 of NNV infection in four tissues of fish reared at 30 kg/m^3^ density was subsequently explored through a transcriptomic study in order to clarify the coordinated response of the viral target tissue (the brain) and the three immune tissues: the head kidney, the spleen, and the liver. As genetic data from shi drum are extremely limited, we firstly performed *de novo* transcriptomic profiling for this species, obtaining a mean of 39,392,132.3 ± 6,007,761.71 clean reads for each sample and a total annotated unigenes of 157,126 (**Supplementary Figures S1–S3**). Almost half of these (41.3%) were homologous to *Larimichthys crocea*, a Sciaenidae fish similar to shi drum (**Figure 2A**). We next compared the identified genes in each tissue in the mock-infected (**Figure 2B**) and NNV-infected (**Figure 2C**) conditions and found that all tissues co-expressed the same number of genes in both conditions, with 15,957 and 15,963, respectively. However, the genes specifically expressed in each tissue were altered upon infection depending on the tissue. Thus, the genes specifically expressed in the head kidney and liver increased when those expressed in the spleen and brain decreased (**Figures 2B, C**). Interestingly, the genes co-expressed in the spleen and brain and in the liver and brain decreased, while the genes co-expressed in the spleen and liver, the spleen and head kidney, the brain and head kidney, and the liver and head kidney increased upon infection (**Figures 2B, C**). Analysis of the DEGs in the tissues between the mock-infected (control) and NNV-infected conditions observed that the number of upregulated genes was higher in all comparisons than the downregulated genes, with the exception of the mock- vs. NNV-infected brain (C-Br vs. NNV-Br) and the NNV-infected brain vs. NNV-infected head kidney (NNV-Br vs. NNV-HK) in which the number of downregulated genes was higher (**Figure 2D**). Looking at the expression profiles and comparing the mock- and NNV-infected profiles in each tissue and in all four tissues analyzed together, the expression profiles of the head kidney, spleen, and brain and the general profiles of the control and infected fish were clustered together, while the liver had a different profile (**Figure 2E**). In general, there were gene expression differences between the mock-infected and infected conditions, but to a lesser extent than between tissues at the same condition (**Figures 2D, E**).

**Figure 3:**
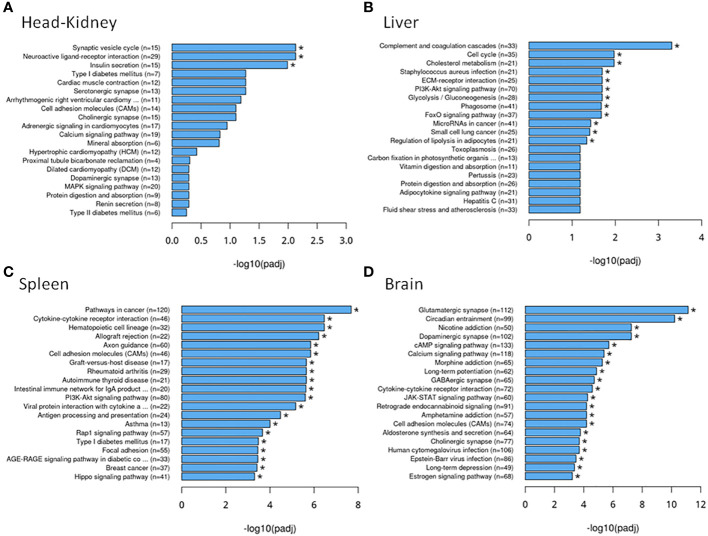
Top 20 Kyoto Encyclopedia of Genes and Genomes (KEGG) pathways associated with the differentially expressed genes in each tissue: **(A)** Head kidney. **(B)** Liver. **(C)** Spleen. **(D)** Brain. * Asterisks denote KEGG pathways significantly modified upon infection.

**Figure 4:**
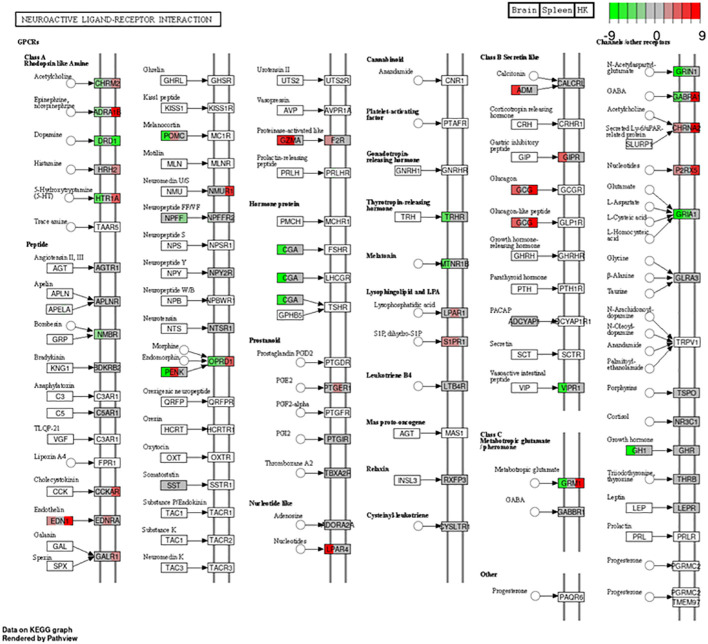
Differentially expressed genes in the neuroactive ligand–receptor interaction pathway (hsa04080) plotted using Pathview (57). Each *gene square* was divided into three parts corresponding to the brain, spleen, and head kidney expression from left to right. Red represents upregulated genes, while green represents downregulated genes.

**Figure 5:**
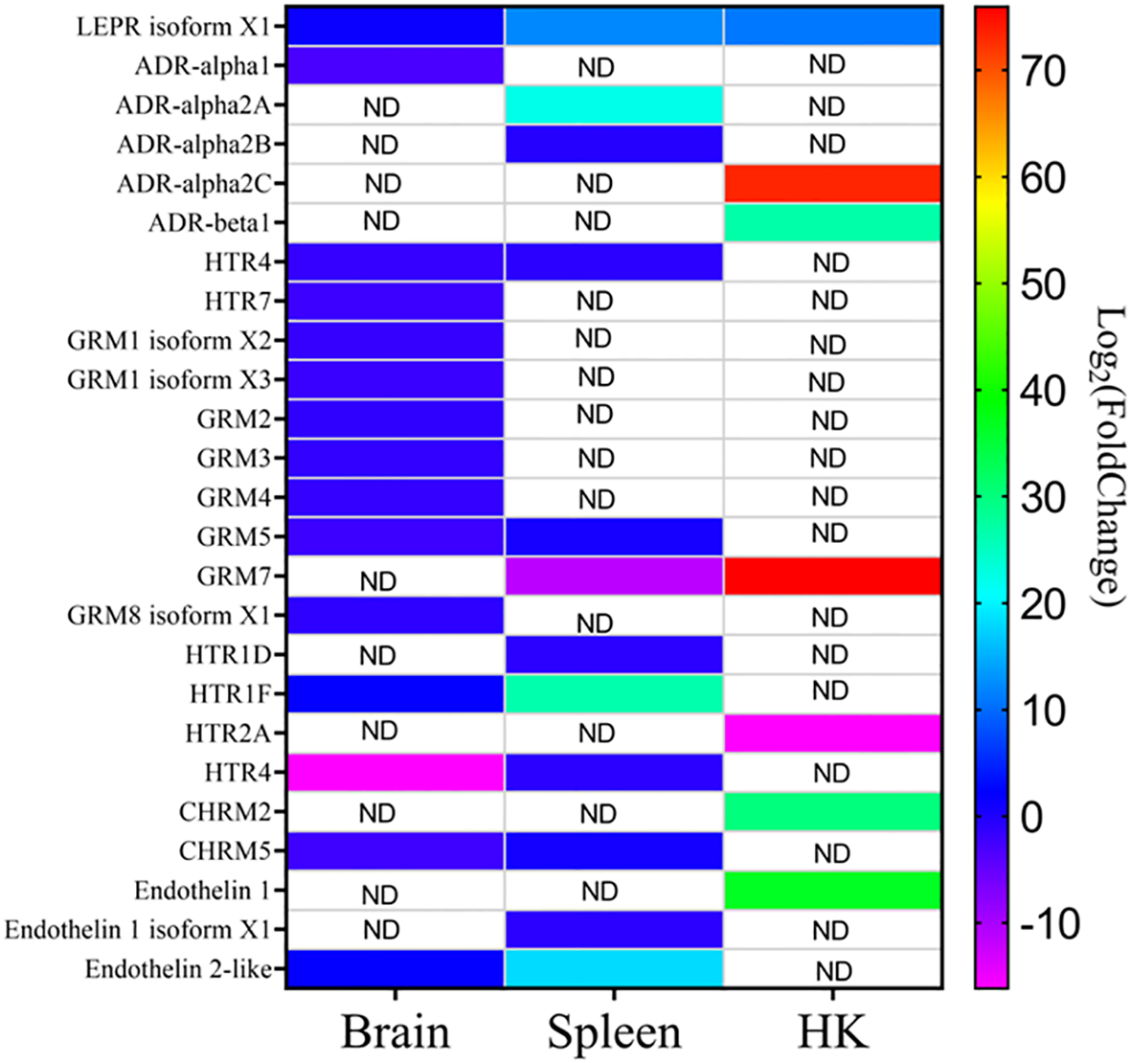
Heatmap showing the different expression of CHRM (muscarinic acetylcholine receptor), HTR (serotonin receptor), GRM (muscarinic glutamate receptor), and LEPR (leptin receptor), endothelin, and their isoforms in the different tissues upon nervous necrosis virus (NNV) infection at stressed conditions. ND, not detected.

Focusing on the functions of the DEGs, in the head kidney (**Figure 3A**), only three KEGG pathways were significantly modified upon infection (the neuroactive ligand–receptor interaction, the synaptic vesicle cycle, and the insulin secretion pathways), while in the liver (**Figure 3B**), there were 12 pathways related to autophagy, phagosome, cholesterol and lipid metabolism, cell cycle and renewal, and innate acute response through complement pathways. In the spleen, 62 pathways were modified, while there were 84 in the brain, the target tissue of NNV (**Supplementary Data 1**). In the spleen (**Figure 3C**), the top 20 pathways significantly modified were related to autophagy and the main spleen function as a secondary immune tissue, such as cytokine–cytokine interaction, hematopoietic cell linage, antigen processing and presentation, migration and cell adhesion, and different signaling pathways. In the brain (**Figure 3D**), however, the top 20 pathways significantly modified were related to cell adhesion, synapses, and hormone signaling (**Figure 3D**). Interestingly, a unique pathway was observed to be modified upon infection in the head kidney, spleen, and brain: the neuroactive ligand–receptor interaction pathway. This pathway was highly modified in the head kidney (29 of the 281 expressed genes were modified, 10%) and, to a lesser extent, in the spleen (54 of the 933 expressed genes were modified, 0.05%) and the brain (117 of the 2,087 expressed genes were modified, 0.05%). Focusing on this pathway (**Figure 4**, **Supplementary Data 2**), most of the receptors altered are involved in neurological disorders and inflammation; however, some of them regulate the vascular system, the feeding behavior and energy consumption, the renewal of cells, and the stress response. The receptors altered in the three tissues were the muscarinic acetylcholine receptor (CHRM), adrenergic receptor (ADR), serotonin receptor (HTR), muscarinic glutamate receptor (GRM), and leptin receptor (LEPR), while the ligands included endothelin and different ligands of the proteinase-activated like receptor (**Figure 4**). Interestingly, multiple receptors of each type with different isoforms in some cases were present in shi drum tissues and were regulated in a tissue-specific manner upon NNV infection at stressed conditions induced by high rearing densities (**Figure 5**).

## 4 Discussion

VER disease is caused by NNV infection, but the clinical signs and susceptibility observed for each species depend on the biological cycle stage and the physiological status of the specimen (21). On the one hand, acute or chronic stress has been associated with higher mortalities during NNV outbreaks (29). On the other hand, there is evidence demonstrating that the mortalities from NNV infection are directly related to the intensity of the inflammatory response associated with the infection (58–61) and inversely to the bw (18, 19, 21, 30). However, and differently from mammals, antiviral treatments that help overcome the exacerbated immune responses and that lead to mortalities upon virus infection have not been developed in fish aquaculture as palliative treatments. This could be due to the lack of knowledge on the immune–neurocrine interactions that orchestrate these effects upon infection. In this framework, we analyzed the growth, immune status, and stress response of juvenile shi drum specimens (30.7 ± 3.10 g bw) under three stocking densities (2, 15, and 30 kg/m^3^) for 27 days and subsequently challenged them with NNV. The main aim was to demonstrate the association of poor welfare and stressed conditions with high NNV susceptibility, as well as the molecular pathways involved.

The optimal density for shi drum culture is unknown as, to our knowledge, this species is not farmed and human consumption is only locally appreciated, but is based on wild fisheries. We routinely rear shi drum at 9–15 kg/m^3^ because it does not show unbalanced behavior and the industrial rearing densities for other Mediterranean fish species are normally around 15 kg/m^3^. Three stocking densities—one very low (2 kg/m^3^), one medium (15 kg/m^3^), and one close to our typical culture density, a high one (30 kg/m^3^)—were tested in order to compare them and to assess whether the stocking density could be a factor that triggers sudden outbreaks. Firstly, we determined by direct observation that shi drum specimens reared at 15 and 30 kg/m^3^ display dark skin and a more nervous behavior than those reared at 2 kg/m^3^, which showed a clear gray color similar to the color of the tank. Interestingly, at medium and high densities, all fish showed a darker coloration, probably due to the fact that they imitate each other instead of the tank. Hence, the density appeared to affect the mimicry of the fishes, changing from a protective resemblance (fish adopt the color of the tank) to a social mimicry (they mime the color of each other), as reviewed (62). In any case, skin color regulation has been known to be linked to stress hormones such as somatolactin, cortisol, and prolactin or thyroid hormones and is a clear sign of stress (63–66). In fact, those fish with dark skin showed the highest levels of serum cortisol from day 7 onwards. Similar to our results, other fish species reared at high densities showed higher levels of cortisol than those reared at a low density (14, 36, 67–75). Taking into account that the study and standardization of a large number of OWIs will allow better monitoring of the health and welfare of fish (21), and that our serum data provided evidence that shi drum specimens show signs of a stress response when reared at densities of 15 and 30 kg/m^3^, we next explored the possibility of identifying noninvasive OWIs by analyzing the levels of cortisol in the water and skin mucus and the levels of glucose and lactate and the total antioxidant activity (ABTS levels) in the skin mucus. For water cortisol, we succeeded in detecting it, but failed to observe differences on day 7 or day 21, contrary to what has been described in rainbow trout where the water cortisol levels significantly changed depending on the density culture conditions (69). Glucose and lactate levels are indicators of stress and are frequently used to evaluate the welfare of farmed fish (14, 36, 70). Thus, in the skin mucus, the cortisol and glucose levels increased in the fish reared at medium and high densities on days 7 and 27 or on day 27, respectively, but no differences were observed on day 21. Although further studies are needed, our data point to the feasible use of stress-related parameters in the skin mucus as OWIs for shi drum. On the other hand, the exceptive negative relationship between stress and growth described in other fish species (76) was partially observed in shi drum. Thus, the SGR index decreased in the fish reared at medium and high densities, but all specimens showed similar use of feed, as revealed by the similar CF values. All of these data demonstrate that mainly fish at 15 and 30 kg/m^3^ showed a poor welfare status, although the effect of the stressed conditions was not excessively substantial. Taking into account that high stocking densities can negatively affect the general welfare of fish (13, 14, 36) by reducing the immune functions (70), we next determined whether the innate immune responses were unbalanced in our stressed fish by analyzing the innate activities in the serum and skin mucus. The fish skin mucus is one of the most important components of the first line of defense against a broad spectrum of pathogens (37, 40, 77) and acts as a barrier between fish and their environment (77), protecting them against microbial infections (37). In this study, no differences were observed in the serum or skin mucus peroxidase, protease, or total bactericidal activity of fish farmed at different stocking densities at any assayed time. However, in the case of antiprotease activity in the serum, fish farmed at medium and high densities for 27 days showed a decrease compared to those farmed at the lowest density; in the skin mucus, the opposite results were observed on day 7. Similarly to the serum antiprotease, the skin mucus lysozyme activity of fish farmed at medium and high densities for 27 days showed a decrease compared with fish farmed at the lowest density. In contrast to other fish species previously studied (73, 78–81), the shi drum specimens reared at different densities did not show differences in the serum lysozyme activity. Taken together, these data suggest that the shi drum skin mucus could reflect more rapidly the immunosuppression triggered by the stress response than the serum, although further studies are needed to clarify this issue. In conclusion, all of the stress parameter and immune data obtained in this study suggested that shi drum specimens reared at 15 and 30 kg/m^3^ display stress responses at the moment of infection (27 days) and that their innate immune responses are not highly unbalanced, at least at naive conditions.

Taking into account the observed susceptibility of shi drum to NNV and the previously described relationship between stress and increased NNV susceptibility observed in other fish species (30), we subsequently studied the effect of NNV infection on shi drum specimens farmed at different stocking densities. Therefore, after being reared at different stocking densities for 27 days, we challenged the fish from each experimental group with the RGNNV genotype (the most common genotype in the Mediterranean area). A group of fish from the highest density group were mock-infected (control group), but no mortalities due to the handling conditions were observed despite the fish having a poor welfare status. In the infected groups, however, only the fish reared at 15 and 30 kg/m^3^ suffered mortalities upon infection, with the mortalities observed in the group reared at the highest density being statistically significant. In addition, this group also recorded the highest cortisol levels and the lowest glucose levels prior to infection. Interestingly, when analyzing the serum and skin mucus parameters of all fish on day 4 of infection by comparing them with the levels displayed by mock-infected fish, no statistically significant differences were observed in all of the parameters analyzed in the group recording no mortalities (2 kg/m^3^). Therefore, in shi drum and under our experimental conditions, we were not able to identify any serum or skin mucus parameter that might be useful in the prediction of the stress occurring upon NNV infection in asymptomatic specimens.

Interestingly, shi drum specimens at the larval stage or with a low bw are very susceptible to RGNNV (5, 9); however, in this study, fish with a bw of 30 g and were reared at low density (2 kg/m^3^) became asymptomatic, even after demonstrating infection, as shown by the expression of the viral CP in their tissues. The inverse relationship between susceptibility to NNV and bw observed in this work has already been described in many species (18). However, to our knowledge, this study is the first to demonstrate the link between stressed conditions and the appearance of VER disease, as well as mortalities in fish with a bw at which they should be asymptomatic.

Shi drum specimens reared at the lowest density (2 kg/m^3^) and infected with NNV showed a high variability in the CP transcription levels in the viral target tissues (the brain and eyes) at both time points analyzed. This variability was strikingly higher for fish reared at 2 kg/m^3^ when compared with those at 15 or 30 kg/m^3^ rearing conditions. Interestingly, although they showed higher variable viral levels, they were asymptomatic and were able to control the disease. It has been recently proposed that an exacerbated inflammatory response leads to mortalities upon NNV infection (58–61). Thus, it is possible that the shi drum specimens at low densities show lower clinical signs and mortalities due to a low inflammatory response, which will likely depend on their physiological welfare. In contrast, the fish reared at high densities might display a high inflammatory response due to their stressed condition. Although further studies are necessary to clarify this issue, our transcriptomic data supported this hypothesis as the pathways related to cytokine and chemokine signaling and inflammation were upregulated in the brain and spleen of the NNV-infected specimens reared at 30 kg/m^3^ (the highest density). In fact, for the immune response, the transcriptomic profile of the spleen showed altered related pathways such as autophagy, antigen processing and presentation, and cytokine interaction, as previously described in an NNV-infected larva transcriptome (82). In addition, the profile observed in the brain resembles similarities to that in a transcriptomic study performed in a brain-derived cell line infected with NNV (83). Interestingly, in the shi drum brain, the estrogen signaling pathway was altered upon infection, as also occurred in European sea bass (84). However, and in contrast to that in European sea bass (85), in the shi drum brain, none of the kisspeptin-regulated genes were altered, nor the gonadotrophin-releasing hormone genes. As these genes belong to the hypothalamic–pituitary–gonadal (HPG) axis, the differences observed between species might modify their capabilities of vertically transmitting the virus. In fact, NNV colonized the gonad of the European sea bass and gilthead seabream specimens upon infection, but it is extremely difficult to detect the virus at 15 dpi as viral gene expression is under the qPCR detection limit, as previously demonstrated (84). However, in shi drum, the expression of the CP gene at 18 dpi was low, but was easily detected in the gonad in all experimental groups (both in fish that showed VER clinical signs and those that did not) using qPCR, as demonstrated in this study. These data led to considering the possibility of developing a methodology to detect NNV in asymptomatic shi drum through gonad biopsies. Nevertheless, further studies are required to clarify the kinetics of the viral transcription rates in surviving fish through time and the relationship between the CP transcription levels in the gonad and the regulation of the HPG axis. This knowledge would be of great importance in combating vertical and horizontal transmission from asymptomatic fish.

In contrast to previous transcriptomic studies that focused on immune responses (82, 83, 86), this work also attempted to understand the molecular pathways involved in the association between stressed or poor welfare conditions and the exacerbated inflammatory response that triggered higher mortalities. In that sense, this work identified for the first time several receptors and ligands belonging to the neuroactive ligand–receptor pathway that were heavily regulated in a systemic way, as their expression was modified in three of the four tissues analyzed. According to our data, the molecules regulated in the three tissues were CHRM, ADR, HTR, GRM, and LEPR. All of these are neurotransmitter receptors in the brain, but are also present in different types of immune cells and other tissues as they belong to the endocrine system (16, 87–91). Therefore, they are a direct link between neurological disorder, endocrine regulation, and immune response. Our data revealed that shi drum expressed all these receptors in multiple isoforms and that they were differentially regulated upon NNV infection in stressed conditions depending on the receptor type, the isoform, and the tissue. The presence of multiple genes that produce an array of receptors differentially expressed in different cells or tissues is expected due to the multiple genome duplications that have occurred during teleost fish evolution (92). However, the complexity observed in the regulation in multiple tissues of these receptors upon NNV infection at stressed conditions points to their importance in orchestrating the imbalance that leads to mortalities upon infection and highlights these receptors as potent targets for pharmacological treatment. Thus, further studies focusing on the neuroactive ligand–receptor pathway are needed to understand the pathology of NNV and its link to poor welfare culture conditions.

## 5 Conclusions

This work is the first study in which the effect of a combination of stocking densities and viral infection in shi drum has been analyzed. In this study, juvenile shi drum specimens (30.7 ± 3.10 g bw) tolerated high rearing densities, even with a poor welfare status. Despite the serum and skin mucus innate immune responses and the antioxidant system being almost unaffected by the poor welfare status and the fish being big enough to be asymptomatic, mortalities occurred upon NNV infection. These data demonstrate that chronic stress due to a high stocking density is a key factor in the pathogenesis of NNV in shi drum juveniles. In addition, our data showed that the skin mucus might reflect more rapidly the immunosuppression triggered by stress response than the serum in shi drum juveniles. Further studies are needed in this sense to clarify its potential for noninvasive sampling to detect stress. Regarding the molecular pathways orchestrating the link between stressed conditions and NNV susceptibility, it was found that, in general, the immune–neuroendocrine system might be crucial and that, in particular, the receptors CHRM, ADR, HTH, and LEPR might be involved in a systemic regulation that could lead to an exacerbated inflammatory response upon NNV infection, causing mortalities at an unexpected fish weight.

## Data availability statement

The data presented in the study are deposited in the NCBI’s BioProject repository, accession number ID 1033413 (https://www.ncbi.nlm.nih.gov/bioproject/PRJNA1033413).

## Ethics statement

The animal study was approved by The Bioethical Committees of the IEO (REGA code ES300261040017) and the approval of the Ministry of Water, Agriculture and Environment of the Autonomous Community Region of Murcia (Permit Number A13211203). The study was conducted in accordance with the local legislation and institutional requirements.

## Author contributions

JG-B: Formal analysis, Investigation, Methodology, Visualization, Writing – original draft. CJ: Data curation, Formal analysis, Investigation, Methodology, Validation, Visualization, Writing – review & editing. MA: Conceptualization, Investigation, Methodology, Resources, Writing – review & editing. AC: Investigation, Methodology, Writing – review & editing. MP: Conceptualization, Funding acquisition, Project administration, Supervision, Visualization, Writing – review & editing. EC-P: Conceptualization, Formal analysis, Investigation, Methodology, Project administration, Resources, Supervision, Visualization, Writing – original draft, Writing – review & editing.
