# Supplementary material for: The susceptibility of shi drum juveniles to betanodavirus increases with rearing densities in a process mediated by neuroactive ligand–receptor interaction
Source: Front Immunol. 2024 Jun 12;15:1304603. doi: 10.3389/fimmu.2024.1304603 (PMC11200141; doi:10.3389/fimmu.2024.1304603)
Supplement: Supplementary file 1 [file DataSheet_1.docx]

Supplementary Material

# Supplementary Data

**Supplementary data 1**. KEGG enrichment pathways significant modify upon infection in the four tissues analyzed.

**Supplementary data 2.** The significant DEG found in the neuroactive ligand-receptor interaction pathway in brain, head-kidney and spleen.

# Supplementary Figures and Tables

## Supplementary Figures

**Supplementary Figure 1.** Gene functional annotation. (**A**)**.** Venn’s diagram mapped with 5 selected database annotation results from 7 data base used. (**B**)**.** KEGG classification.

**Supplementary Figure 2**. Gene functional annotation, Go classification.

**Supplementary Figure 3.** Gene functional annotation, KOG Classification.

**Supplementary Figure 4.** Replicates comparison of the RNA-Seq data. (**A**)**.** A Heatmap of the Pearson correlation coefficient between samples showing smaller differences between fish replica than between tissues or conditions. (**B**)**.** Validation of the RNA-seq data by means of the qPCR. Each dote represents the mean value for each selected gene. A linear regression of the data was performed

## Supplementary Tables

.**Supplementary Table 1.** Primer sequences used for gene expression analysis by qPCR.

| **Target gene** | **Accession number** | **Forward primer (5´3´)** | **Reverse primer (5´3´)** |
| --- | --- | --- | --- |
| NNV coat protein (CP) | D38636 | CAACTGACAACGATCACACCTTC | CAATCGAACACTCCAGCGACA |
| β-actin (actb) | AJ493428 | TCTTCCAGCCATCCTTCCTCG | TGTTGGCATACAGGTCCTTACGG |
| somatolactin (sl) | Cluster-48508.10770 | TTCTGCAGGTAGGCCAAAGG | CCATCCCCAGCTCCAAAAGT |
| pro-opiomelanocortin (pomc) | Cluster-48508.24625 | CGTTCTTCCAAAAACGACCCA | ATTGTCTGTATCGACTAAGCGGT |
| growth hormone precursor (gh) | Cluster-48508.8457 | ACCTACCTGACGGTGGCTAA | GCAGAGTCAGAGAGCGTGG |
| trypsin-3 isoform X1 (try3) | Cluster-48508.19957 | AGGATTTTGGCGGTCACAGT | GCGGATGCTGTATATGGCCT |
