# Supplementary figures and images for: The susceptibility of shi drum juveniles to betanodavirus increases with rearing densities in a process mediated by neuroactive ligand–receptor interaction
Source: Front Immunol. 2024 Jun 12;15:1304603. doi: 10.3389/fimmu.2024.1304603 (PMC11200141; doi:10.3389/fimmu.2024.1304603)

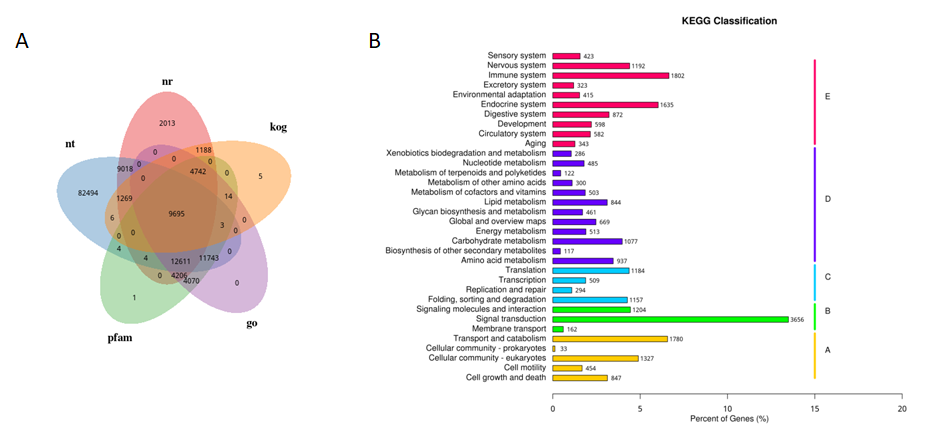

Supplement: Supplementary file 2 [file Image_1.tif]

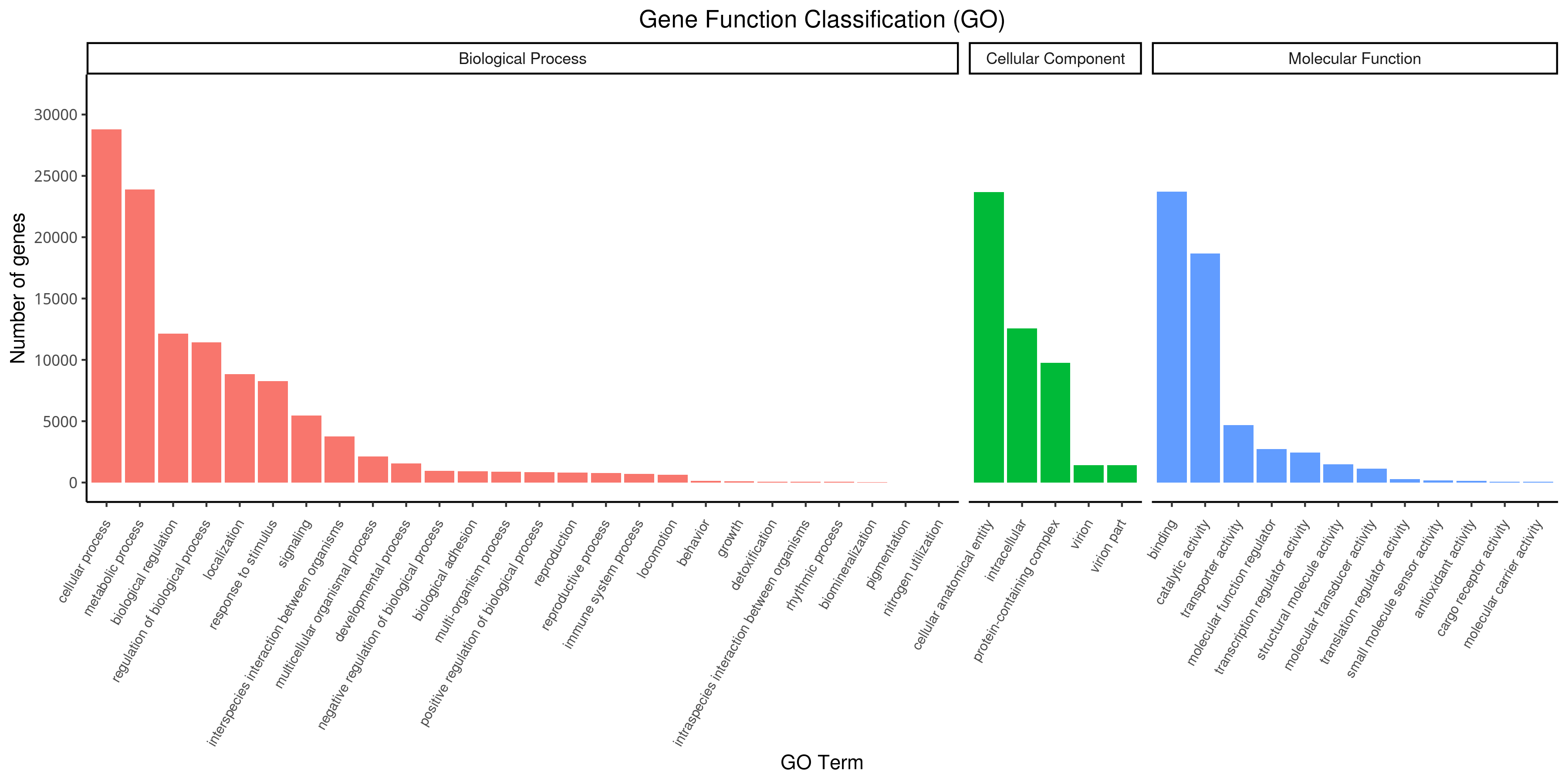

Supplement: Supplementary file 3 [file Image_2.tif]

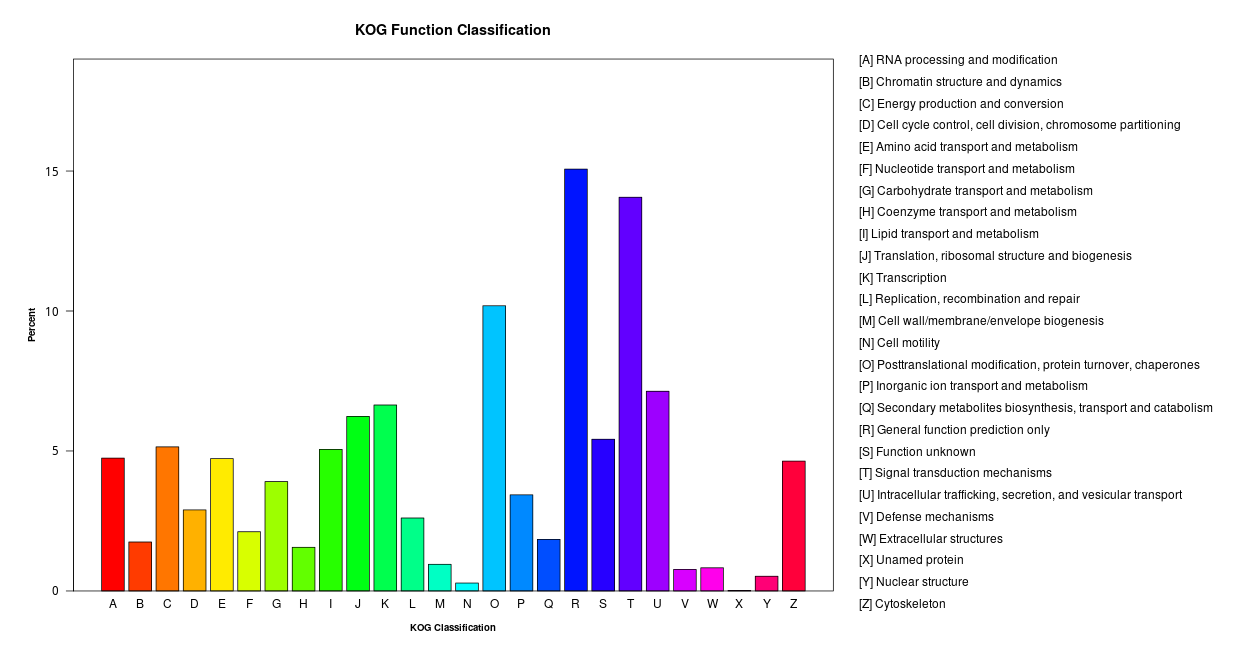

Supplement: Supplementary file 4 [file Image_3.tif]

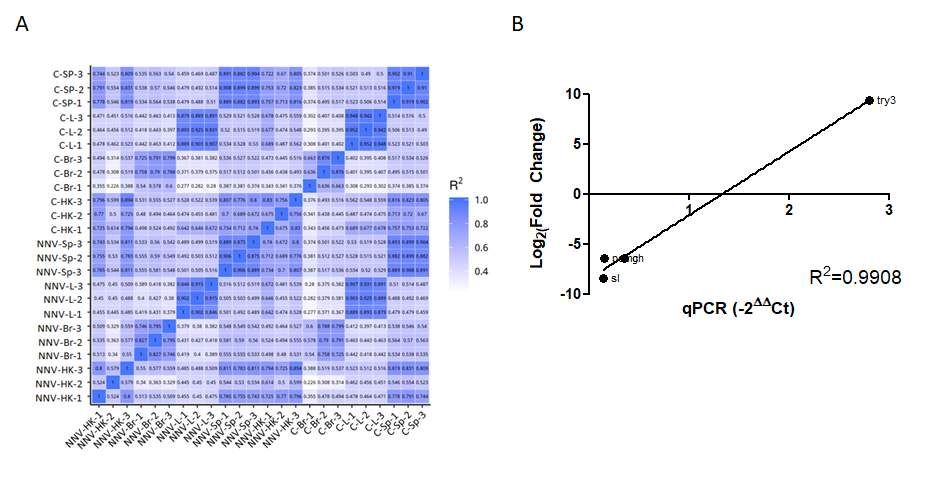

Supplement: Supplementary file 5 [file Image_4.tif]
